# Prediction of future cognitive impairment among the community elderly: A machine-learning based approach

**DOI:** 10.1038/s41598-019-39478-7

**Published:** 2019-03-04

**Authors:** Kyoung-Sae Na

**Affiliations:** Department of Psychiatry, Gil Medical Center, Gachon University College of Medicine, Incheon, Republic of Korea

## Abstract

The early detection of cognitive impairment is a key issue among the elderly. Although neuroimaging, genetic, and cerebrospinal measurements show promising results, high costs and invasiveness hinder their widespread use. Predicting cognitive impairment using easy-to-collect variables by non-invasive methods for community-dwelling elderly is useful prior to conducting such a comprehensive evaluation. This study aimed to develop a machine learning-based predictive model for future cognitive impairment. A total of 3424 community elderly without cognitive impairment were included from the nationwide dataset. The gradient boosting machine (GBM) was exploited to predict cognitive impairment after 2 years. The GBM performance was good (sensitivity = 0.967; specificity = 0.825; and AUC = 0.921). This study demonstrated that a machine learning-based predictive model might be used to screen future cognitive impairment using variables, which are commonly collected in community health care institutions. With efforts of enhancing the predictive performance, such a machine learning-based approach can further contribute to the improvement of the cognitive function in community elderly.

## Introduction

Cognitive impairment has devastating effects on individuals, caregivers, and society. Individuals with cognitive impairment frequently suffer from comorbid psychiatric conditions (e.g., depression, wandering, agitation, insomnia, psychotic symptoms, etc.)^1,2^. It is commonly associated with physical diseases, such as diabetes mellitus (DM) and cardiovascular diseases^3^. Individuals with cognitive impairment also experience a decreased quality of life^4^.

The harmful effects of cognitive impairment are not restricted to its advanced forms such as dementia. In addition to the well-known risk of progress to dementia^5^, mild cognitive impairment (MCI) can also cause substantial psychological symptoms in caregivers^6^ and patients^7^. The prevalence of MCI is 10–20% among the elderly. Approximately 30–40% of cases with MCI consequently progress to dementia^8^. The financial burden and medical complications among patients with MCI are certainly higher than those for healthy individuals^9^.

Currently, the best way to prevent or minimize this devastating course is to detect risk in people early and begin intervention^10^. Many researchers have identified neurobiological, genetic, and neuroimaging biomarkers for cognitive impairment, particularly in Alzheimer’s disease^10,11^. These efforts should persist, and would consequently yield results. However, the high costs of neuroimaging and genetic evaluation restrict their wide dissemination to the community elderly.

Various factors, including sociodemographic, personal, health, and quality of life, contribute to future cognitive functions^12–15^. These factors provide invaluable information that is not captured by a simple cognitive test, such as the Mini-Mental Status Examination (MMSE). For example, regular exercise has therapeutic effects for stress-induced cognitive impairment^16^. If one exercises regularly, then he or she is likely to have an advantage in terms of cognitive functioning. Alcohol use and depression are well known for their adverse effects on cognitive functions^17,18^. However, simply identifying the presence or absence of various risk or protective factors is not helpful in predicting future cognitive impairment. These variables can be meaningful when their complex interactions are analyzed using appropriate algorithms.

This study sought to build a predictive model that incorporates variables that can be easily obtained at a low cost. Machine learning is used to integrate these variables and construct a reproducible predictive model.

## Results

### Participant data

Table 2 summarizes the variables used in the predictive model. The mean (SD) age of the participants at baseline was 70.4 (6.97) years. The mean (SD) score on the K-MMSE at baseline was 26.9 (3.14). The mean (SD) K-MMSE score after 2 years was 25.9 (4.33). The number of the elderly with cognitive impairment after 2 years was 80 (2.34%).

**Table 1:** Cut-off point of the scores on the Korean Mini-mental Status Examination according to age group and gender.

Age group	Illiteracy	Uneducated to <5 years	Elementary school (6 years)	More than 7 years
45–64	18	23	25	25
65–69	16	22	23	24
70–74	15	21	22	24
75–84	14	20	21	22
85–90	11	18	19	26

**Table 2:** Summary of the sociodemographic, health, interpersonal, quality of life, and subjective well-being variables.

	Mean or N	SD or %
**Sociodemographic**		
Age	70.4	7.0
Gender, male	1586	46.3
Educational level, ≥high school	1157	33.8
Marital status, married	2622	76.6
**Religion**		
None	1844	53.9
Protestants or Catholic	860	25.1
Buddhism	720	21.0
**Region**		
Metropolis	1438	42.0
Small-medium sized city	1029	30.1
Rural area	957	27.9
Children alive ≥3	2108	61.6
Cohabitating children	1047	30.6
Medical aid, yes	167	4.9
Family members with functional impairment	166	4.9
**Health**		
CES-D, mean	6.03	4.8
K-MMSE, baseline	26.9	3.1
K-MMSE, after 2 year	25.9	4.3
Cognitive impairment after 2 years	80	2.3
Number of chronic disease ≥2	1395	40.7
Psychiatric diseases	134	3.9
Neurovascular diseases	182	5.3
Arthritis	996	29.1
Cardiac disease	357	10.4
DM or hyperglycemia	717	20.9
Hypertension	1614	47.1
Taking regular medicine	2383	69.6
Cancer	206	6.0
Dentures	799	23.3
Admission in recent 2 years	389	11.4
**Alcohol drinking**, **≥1 per week**		
Soju	838	15.2
Beer	555	10.6
Makgeoli	401	8.2
**Drinking habit**		
Normal	2268	66.2
Overdrinking	1022	29.9
Alcoholic	134	3.9
**Smoking**		
None	2324	67.9
Cessation	670	19.6
Smoking	430	12.5
**Functional**		
Regular exercise, ≥1 per week	1273	37.2
Limited everyday activity due to health, yes	1154	33.7
Falls in recent 2 years	75	2.2
Meeting close person, ≥1 per week	2172	63.4
Meeting children, ≥1 per week	798	23.3
Attending social meeting	2248	65.6
Current working, yes	1201	35.1
**Subjective wellbeing**		
Satisfaction in own quality of life	2327	68.0
Satisfaction in own economy	1844	53.9
Satisfaction in own health	2093	61.4

All data are presented as mean (SD) or number (%).

CESD-D: Center for Epidemiological Studies—Depression; K-MMSE: Korean Mini-mental State Examination.

### Performance

**Figure 1:**
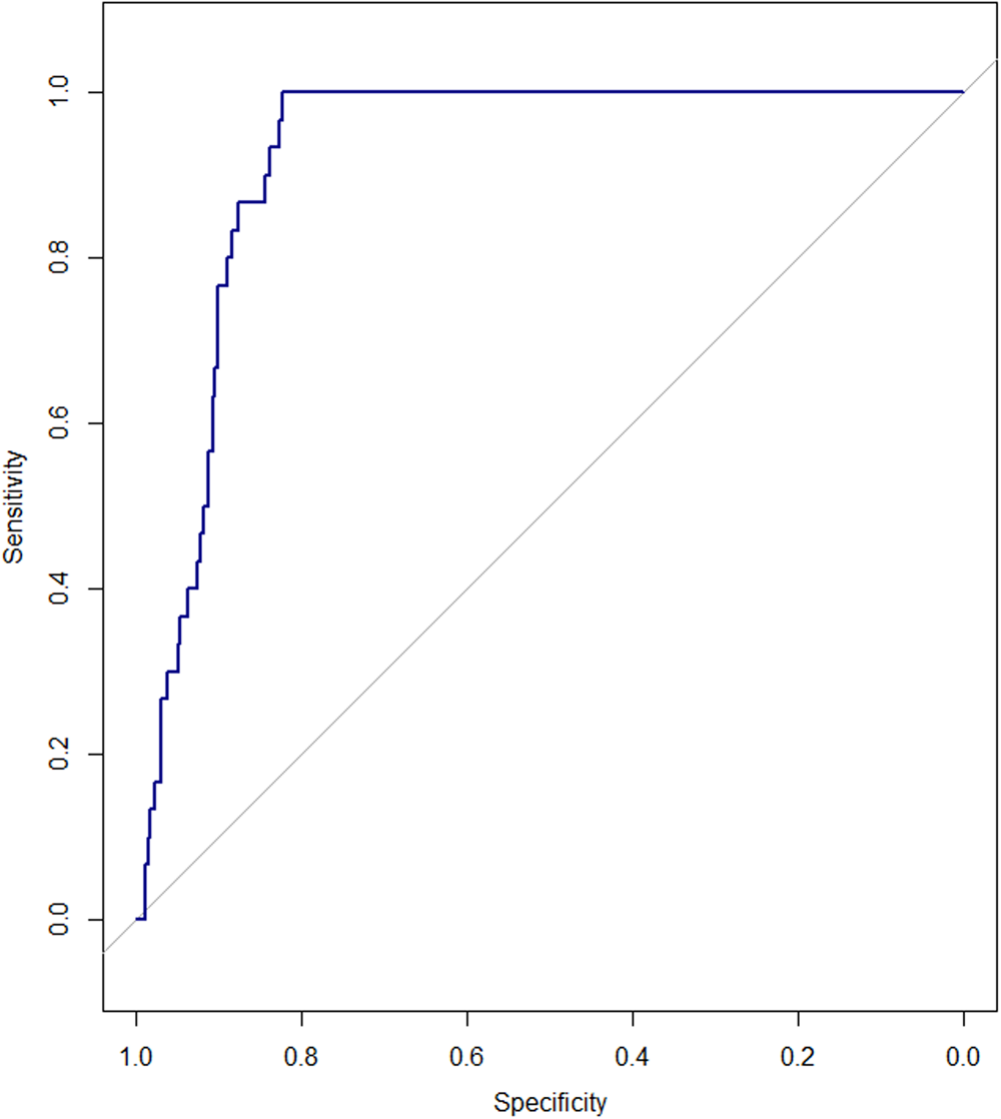
Area under the receiver operating characteristics curve of the gradient boosting machine. The area under the receiver operating characteristics curve (AUC) is 0.921. Sensitivity reaches 1.0 right after specificity decreases below 0.8. This pattern of sensitivity might have arisen from the small number of positive cases (30 out of 1022) in the test set.

**Figure 2:**
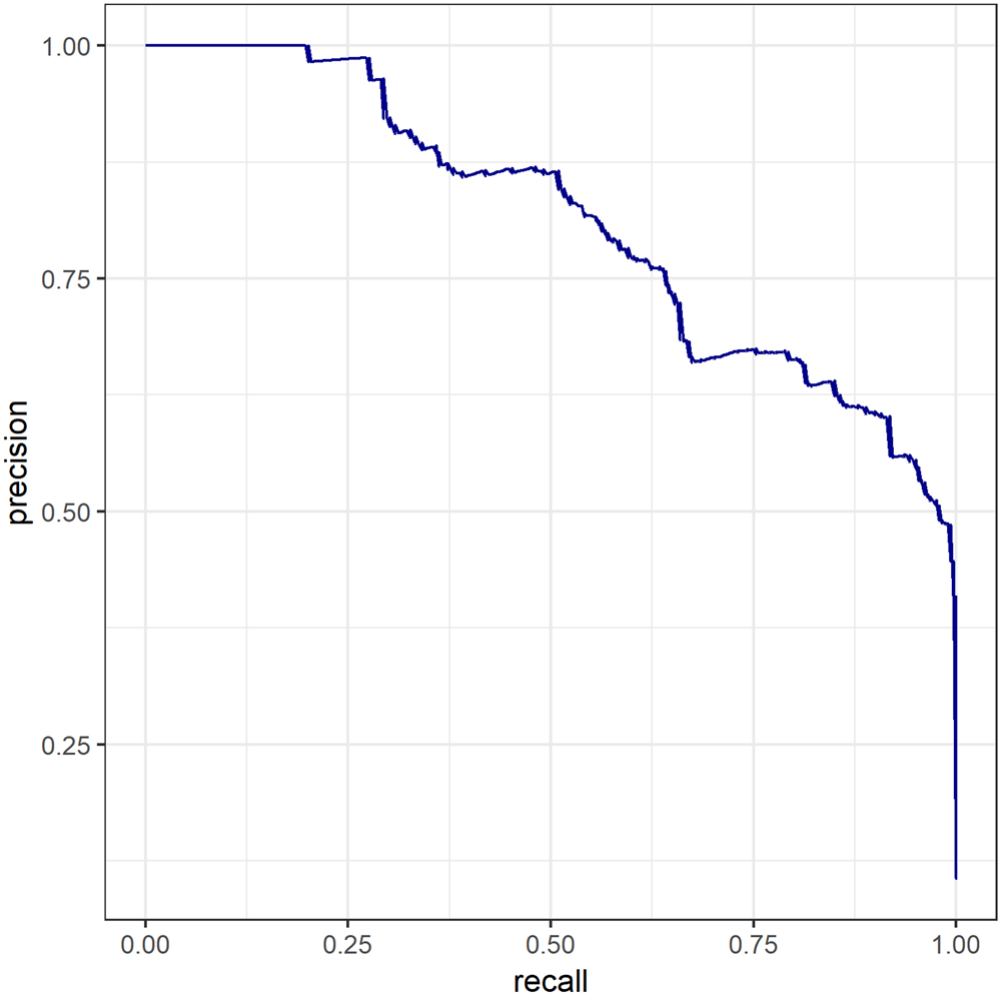
Precision–recall plot of the gradient boosting machine. The precision–recall plot shows that precision is consistently maintained above the prevalence rate of cases. The prevalence rate is too small (0.03); hence, the threshold line is not visualized in the above figure.

Table 3 shows that the sensitivity of the predictive model was excellent (0.967). The negative predictive value (NPV) was 0.999, while precision (positive predictive value) was 0.143. The AUC (0.921) represents good binary classifying performance (Fig. 1). The precision–recall plot shows that the classifier performs well considering the highly imbalanced dataset (Fig. 2).

**Table 3:** Performance metrics of the gradient boosting machine.

Sensitivity	Specificity	MCC	Accuracy	AUC	Precision	NPV	F_1_-scores
0.967	0.825	0.335	0.829	0.921	0.143	0.999	0.249

AUC: area under the receiver operating characteristic curve; MCC: Matthews correlation coefficient; NPV: negative predictive value.

### Importance of variables

**Figure 3:**
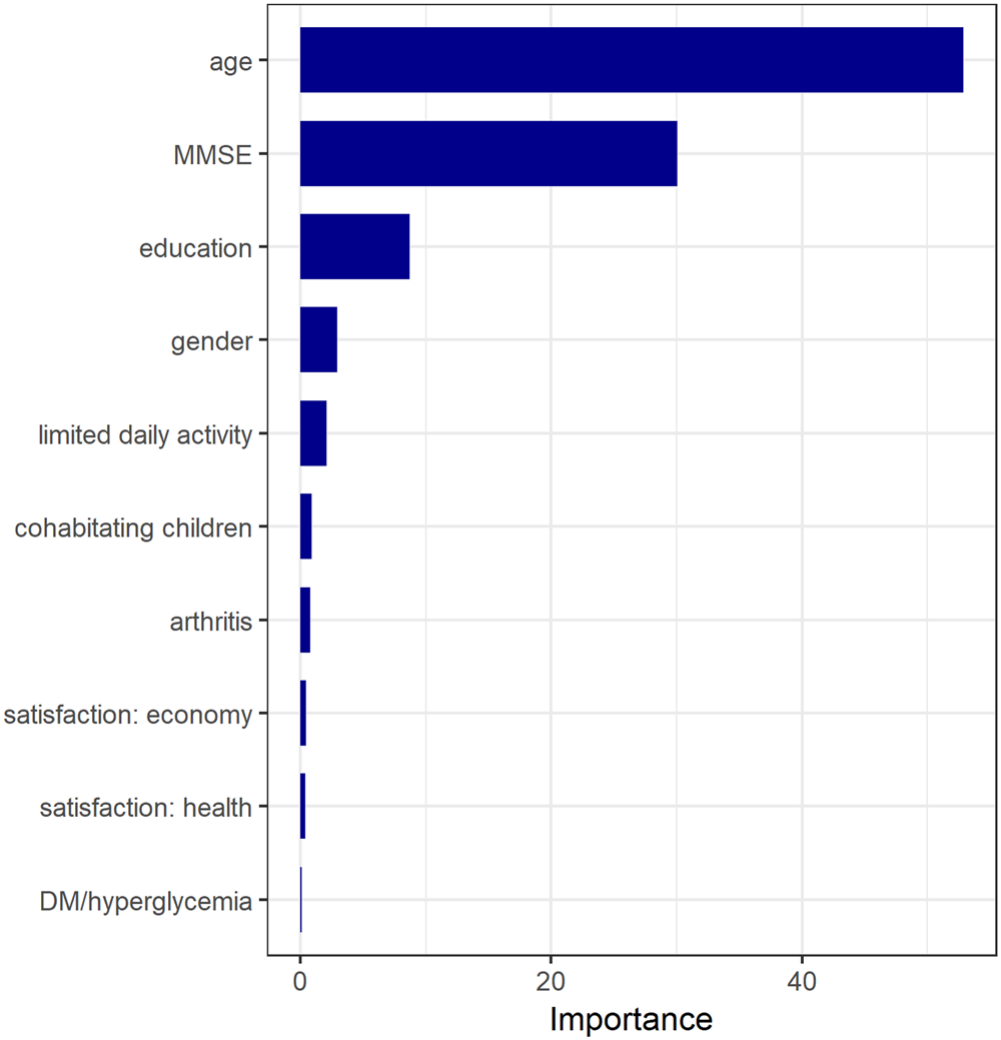
Importance of variables in the gradient boosting machine. After the well-known influential factors for the cognitive function, the limited daily activity caused by health problems is ranked fifth. Cohabitating children, chronic diseases (arthritis and DM/hyperglycemia), and subjective wellbeing (satisfaction in their own economic and health status) are included in the top 10 influential variables in this predictive model.

Figure 3 presents the 10 most influential variables. As expected, age, MMSE, and education levels had the strongest influences on the predictive model. The limited daily activity caused by health problems was ranked fifth, followed by the presence of cohabitating children, arthritis diagnosis, subjective satisfaction in their own economic state, subjective satisfaction in their own general health, and DM or hyperglycemia diagnosis.

## Discussion

A predictive model with machine learning algorithms was built herein to classify elderly at risk for cognitive impairment 2 years later. The predictive model with GBM showed excellent sensitivity (0.968) and AUC (0.921). Specificity (0.825) and accuracy (0.829) were tolerable. Overall, this predictive classifier seemed to have good screening performance^19^. This predictive performance is better than that of the previous study, which used machine learning to compute the likelihood of dementia 1 year later^20^. However, the performance of the predictive model should be cautiously considered in terms of the low F_1_-score and MCC. The low F_1_-score was already expected because the dataset was highly imbalanced in favor of the negative cases. The modest MCC values might have resulted from the low precision (0.143). In short, if 1,000 elderly people are classified to the cognitive impairment group, only 143 would actually be suffering from cognitive impairment. Further, the excellent negative predictive value (0.999) and sensitivity ensure that almost all elderly people classified as having no future cognitive impairments will be actually normal. This high-recall and low-precision predictive model is frequently used in the field of medicine, where failure of detection of the risk group can lead to critical health problems; this is also why the primary outcome measure was set to sensitivity.

The longitudinal approach of this study is differentiated from several studies using neuroimaging modalities. Many of such studies built classification models based on the matched case-control design (for a detailed review, please refer to the study by Pellegrini *et al*.^21^). A similar proportion of the case and controls is advantageous for building a model with stronger performance metrics. However, in the real-world, the number of the elderly with cognitive impairment is substantially lower than those with normal cognitive function. Hence, the proposed algorithm would be suitable for screening future cognitive impairment in practice.

The high cost and restricted measuring environment of MRI and PET are possible limitations of their wide application to community-dwelling elderly. Needle insertion and the use of radioactive materials are additional drawbacks of PET. In contrast, the predictive models in this study only required variables that can be easily collected during the routine practice of the community healthcare centers. Together with good predictive performances, the availability of the variables makes it possible to disseminate and screen future cognitive impairment among community-dwelling elderly.

By contrast, variables that are important in the predictive models should be noted. The importance of the baseline cognitive function, age, and educational levels for future cognitive function has been consistently reported^22,23^. The other major important variables of the predictive model herein were the limited daily activity caused by health problems, presence of the cohabitating children, chronic diseases (arthritis and DM), and subjective wellbeing (satisfaction in their own economic and health status). Although the weights of the variables are relatively small, this supports the notion that there may be complex direct and indirect interactions among various factors on the cognitive function^24^. Previous studies reported a close association between cognitive functions and life satisfaction^25^. Cohabiting children also had beneficial roles in the cognitive functioning of the elderly. First, they can serve familiar relationships in the family, thereby reducing loneliness in the elderly. The elderly frequently experience loss and loneliness. Recent studies have suggested that loneliness can exert harmful effects on the cognitive functions and mental health of the elderly^26^. Children can be a psychological comfort and prevent solitude in the elderly^27^. Additionally, children who frequently meet with their elderly patients can easily recognize any significant changes in their parents’ cognitive functions. This may lead to early evaluation and intervention, which contribute to a better cognitive outcome. However, it is plausible that cognitive impairments would have reciprocal relationships with the quality of life, subjective wellbeing, and functional disability in the elderly^28^.

Although several important factors that contribute to the predictive model have been briefly discussed, what counts is not the individual risk or protective factors, but a model that encompasses such factors and identifies which one is likely to be cognitively impaired. To date, several research groups, not limited to the Republic of Korea, have used the KLoSA data to examine the risk factors of cognitive impairment. One group evaluated the cognitive changes between 2008 and 2012 and identified that baseline social activities, including contact with their children, were associated with less cognitive impairment^29^. Other studies have shown that gender^30^ and body mass index^31^ played a role in the future cognitive functioning among the elderly. Some studies revealed risk factors for the cognitive functioning in a cross-sectional design^13,32,33^. However, although the data similar to those in the previous studies were used herein, the present study differed in terms of the objective. While all the previous studies using the KLoSA data aimed to identify the risk factors for cognitive impairment, this study used data from the national survey to pragmatically build a predictive model.

Several limitations should be noted. First, a binary classifier was built instead of a multiclass classifier (healthy controls vs. MCI vs. dementia). As stated in the Introduction section, finely discriminating the degree of cognitive impairment was not the objective of this study. Rather, this study intended to develop a model that can be widely used among the community-residing elderly given variables that are easy to collect at reasonable costs. Second, the cognitive impairment was measured without clinical diagnostic evaluation. Clinical criteria, such as the Diagnostic and Statistical Manual of Mental Disorders, 5th edition (DSM-5)^34^ and the International Classification of Diseases, 10th edition (ICD-10), must be used to diagnose the severe form of cognitive impairment, such as dementia^35^. Third, we may also need additional measurements, including hematological, urine, and brain MRI to specify the types of dementia. However, most of these professional measurements are taken at the hospital for selected populations who have risk factors and/or symptoms. In contrast, the predictive model for future cognitive impairment was constructed based on the community-residing middle-aged to elderly. The primary objective of this machine learning-based predictive model is to screen the elderly who will likely have cognitive impairment 2 years later, but not confirm the specific neurocognitive disorders. The weakness of the MMSE, varying accuracy according to the age, educational levels, and gender^36^ were minimized by applying stratified cut-off points for each subgroup. Hence, the lack of a clinician-made diagnostic evaluation will not substantially gilt off the strength of this study.

This study demonstrated that the sociodemographic, health, functional, and interpersonal, and subjective domain variables can be used to predict future cognitive impairment among community-dwelling elderly. These variables can be easily collected from the elderly and their close relatives; hence, this predictive model can be widely disseminated to the community. Considering the effort put into enhancing the performance of this predictive model, the model can be of help to community-dwelling elderly in terms of promoting cognitive function before it becomes worse.

## Methods

### Participants and data

Data from the Korean Longitudinal Study of Aging (KLoSA)^37^ from 2014 to 2016 were used. The participants of the survey were recruited using a multistage stratified cluster sampling based on 15 geographical areas and housing types. Blaise (http://blaise.com) was used for convenient and accurate data collection. Blaise is a computer-assisted personal interviewing software widely used over 30 countries. A skilled interview is important for obtaining reliable information; hence, intensive education and mock interviews were conducted 1 month before the start of the survey. All participants provided written informed consent before the data collection.

The sampling frame of the KLoSA was initially created and used in the population census in the Republic of Korea in 2005^38^. The first survey was conducted between August and December in 2006. The initial respondents were 10,254 individuals aged over 45 years. The KLoSA survey is biennially performed. The author used data from 2014 (wave 5) and 2016 (wave 6) to exclude the very young age group and utilize the most recent information. Based on the previous study^39^, the criteria of the cognitive impairment were defined as the Korean Mini-mental State Examination (K-MMSE) scores below 1 standard deviation of the mean scores of age by educational level stratified groups (Table 1). Unlike the original study^39^, the current study categorized uneducated and less than 6 years of education into the same group due to the lack of the detailed information on the years of education less than 4 years.

The inclusion criteria at baseline were elderly aged between 60 and 89 without cognitive impairment. The total number of participants included in the final dataset was 3424 (i.e., 1586 males and 1838 females).

Based on previous studies^9,14^ and expert opinions, the author used 35 variables associated with cognitive functions from the four main domains (i.e., sociodemographic, health, functional, and subjective wellbeing) (Table 2).

The study protocol was approved by the Institutional Review Board in the Gachon University Gil Medical Center (GCIRB2018-152). All methods were performed in accordance with the relevant guidelines and regulations.

### Preprocessing

The proportion of the training and hold-out test set was determined as 0.7 and 0.3, respectively. The synthetic minority over-sampling technique (SMOTE) was used to deal with the imbalanced ratio of the elderly with and without cognitive impairment^40^. Unlike up-sampling, which simply replicates duplicate samples, the SMOTE generates artificial data that resemble the original dataset. The SMOTE was only applied to the training set in the cross-validation to avoid any possibility of overfitting. The final performance metrics were evaluated with the hold-out test set, which has never been included in the SMOTE or cross-validation procedures.

Given the number of the observations and variables, no prior feature selection process was conducted. The importance of the variables in each predictive model was separately summarized.

### Machine learning model

All machine learning processes were conducted using the *caret* package^41^ for R (https://www.r-project.org/). The *caret* package enables the construction of a unitary preprocessing dataset and, thus, provides a reliable comparison between different machine learning models. The gradient boosting machine (GBM) was used herein because it utilizes the ensemble approach; hence, the predictive model might be built while minimizing classifying errors. The principles and practices of the GBM are well described in several literatures^42,43^; thus, the essential features of the GBM are only briefly summarized herein. The GBM is an ensemble algorithm with the boosting method based on the decision tree model^44^. The boosting algorithm initially generates a weak classifier with the same weights for all instances. This weak classifier can correctly classify the binary class only slightly more than random classifiers do by chance. The classifying algorithm is then trained again. This time, the weight, which wrongly classified the target in the previous training, is increased, whereas the weight of the correct classifiers is decreased. This adjustment of the weights makes the classifier more robust to the previously misclassified cases. The ‘gradient’ in the GBM has the same meaning as the term ‘gradient descent.’ Gradient descent is one of the several mathematical algorithms by which the boosting methods update the classifier to become stronger. The gradient descent adjusts the parameters to minimize a loss function and determine the optimal point with the smallest error. For example, the fourth classifier is fitted to the residual error made from the third classifier. This process of sequentially adding new weak classifiers with gradient descent is iterated until the classifying performance of the classifier becomes perfect (i.e., the error rate is 0) or the iteration reaches the predetermined number.

### Cross-validation

This *k*-fold cross-validation is a recommended cross-validation method because it can secure more samples for training without loss of sample size as compared to the splitting method^45^. Within the training set, a ten-fold cross-validation was conducted with five repeated processes.

### Hyperparameters

**Figure 4:**
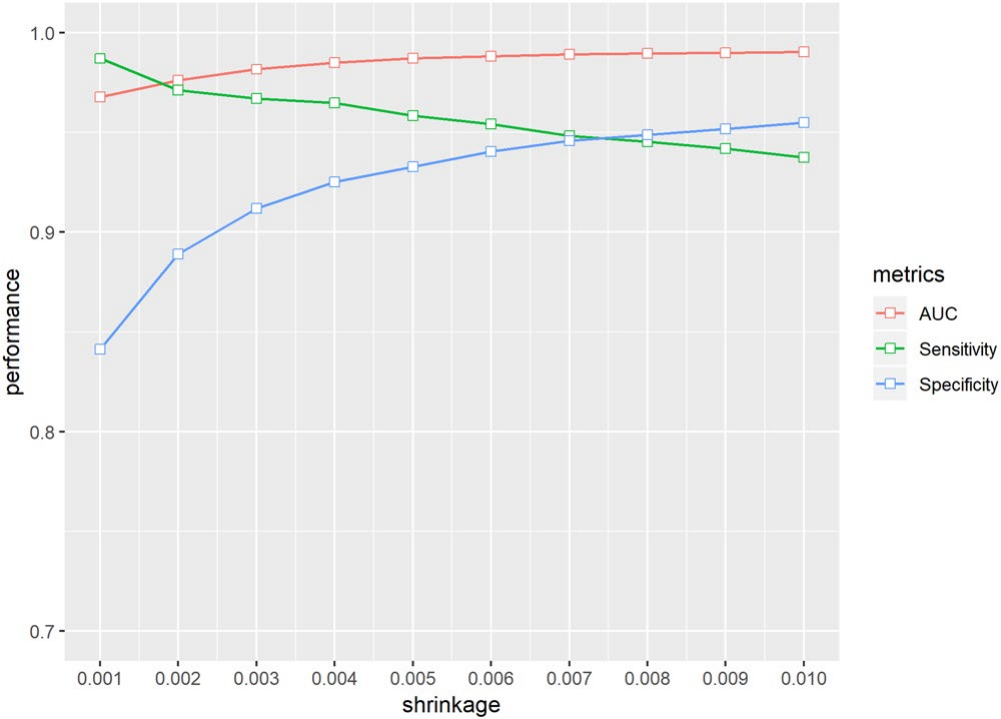
Hyperparameter tuning of the gradient boosting machine. This figure shows the distribution of the area under the receiver operator curve (AUC), sensitivity, and specificity. Specificity increases and sensitivity decreases as shrinkage increases. The AUC performance shows increasing trends along with shrinkage. The optimal shrinkage herein is set to 0.007, in which sensitivity has high values, while specificity does not exceedingly decrease.

Hyperparameters were tuned by the grid search during the cross-validation. The learning rate is the basic component of hyperparameters in most machine learning algorithms. The time to reach the optimal point with the least error can be delayed when the learning rate is too low. However, when the learning rate is too large, the algorithm might jump over the optimal point such that suboptimal points can be obtained after the predetermined length of learning. The depth of trees reflects the number of splits. More interactions among the variables were considered in the algorithm as the depth of trees became large. Finally, the following hyperparameters were tuned: *shrinkage* (learning rate) was 0.007; *n*.*trees* (number of trees) was 1000; *interaction*.*depth* (depth of trees) was 4; and *n*.*minobsinnode* (minimum number of observations allowed in the trees of terminal nodes) was 5. Figure 4 visualizes the performance metrics according to the *shrinkage* values.

### Performance metrics

The performance metrics were considered based on the imbalanced proportion of the elderly with cognitive impairment. Detecting cognitive impairment among a large number of observations is important when applied in real-world practice; hence, sensitivity was first considered. The overall accuracy and the area under the receive operator curve (AUC) were measured as secondary performance metrics.

The F_1_-score and Matthew’s correlation coefficients (MCC) were used as the performance metrics^46^. The F_1_-score was formularized using the true positives (TP), false positives (FP), and false negatives (FN) $(\frac{2TP\,}{2TP+FP+{FN}})$. As the F1-score does not account for the true negatives (TN), it has limited utility in the highly imbalanced data in which majority of the cases belong to the negatives.

In contrast, the MCC utilizes all four major components of the confusion metrics $(\frac{(TP\times TN)-(FP\times FN)\,}{\sqrt{(TP+FP)(TP+FN)(TN+FP)(TN+FN)}})$. The MCC are a discretized form of the Pearson’s correlational analysis; thus, the MCC value is interpreted in terms of the Pearson’s correlational coefficients, *r*^47^. Unlike other performance metrics with a range of 0 to 1, the range of the MCC is from −1 to 1. The value of −1 in the MCC indicates complete disagreement between the actual and predicted values, such as the value of 0 for accuracy. In contrast, the value of +1 in the MCC represents complete agreement between actual and predicted values, such as 1 for accuracy. Although the interpretation of the MCC might not be intuitive as other performance metrics ranging from 0 to 1, it is advantageous over the F_1_-score in the imbalance dataset.

## Data Availability

The dataset generated and analyzed in the current study is available from the corresponding author upon reasonable request. The predictive model is deployed and available at https://ksna19.shinyapps.io/Prediction_of_cognitive_function.
